# Editorial: Modifiable risk factors for chronic kidney disease progression

**DOI:** 10.3389/fendo.2026.1825701

**Published:** 2026-04-10

**Authors:** Simona Musto, Carmine Secondulfo, Serena Migliarino, Carmine Izzo

**Affiliations:** 1 Department of Medicine, Surgery and Dentistry “Scuola Medica Salernitana”, University of Salerno, Baronissi, SA, Italy; 2 Cardiology Unit, University Hospital “San Giovanni di Dio e Ruggi d’Aragona,”, Salerno, Italy

**Keywords:** cardiorenal nexus, cardiovascular disease, chronic kidney disease, modifiable risk factors, renal biomarkers

Chronic kidney disease (CKD) affects more than 850 million individuals worldwide and represents one of the leading causes of cardiovascular morbidity and mortality. Beyond progressive loss of renal function, CKD is increasingly recognized as a systemic disorder driven by metabolic, inflammatory, and vascular mechanisms. Importantly, many of the determinants of CKD progression—including hypertension, diabetes, obesity, dietary factors, and metabolic dysregulation—are potentially modifiable, making early identification and targeted intervention central to reducing the global burden of disease. This Research Topic brings together contributions addressing various aspects of CKD, from global epidemiology to pathophysiological mechanisms, from the identification of emerging biomarkers to the evaluation of new therapeutic approaches.

Several studies in this Research Topic analyse the global burden of CKD and the influence of dietary and metabolic factors on its progression. Li et al.’s analysis of Global Burden of Disease data from 1990 to 2021 shows that CKD remains a major global health problem, with regions of low socio-demographic index (SDI) experiencing the highest levels of disability-adjusted life years (DALYs) (1). Similarly, Lu et al. found that the global burden of CKD attributable to high sodium intake increased between 1990 and 2021, with rises in both mortality and DALYs. Additionally, Huang et al. examined the burden of CKD associated with hypertension attributable to dietary factors, finding a particularly marked increase among older populations and in countries with low SDI. These findings are supported by the study by Wang et al., which indicates that hypertension is an early and increasing determinant of CKD, even in younger populations. Furthermore, another study by Wang et al. showed that CKD associated with type 2 diabetes mellitus is strongly influenced by dietary factors, consistent with the role of diet in modulating oxidative stress and systemic inflammation. Taken together, these findings highlight how the global burden of CKD is increasingly shaped by modifiable lifestyle and metabolic determinants that vary substantially across geographic and socio-demographic contexts. Dietary patterns, sodium intake, and cardiometabolic risk factors appear to act synergistically in accelerating renal dysfunction, particularly in populations undergoing rapid epidemiological transition. These observations reinforce the need for prevention strategies that integrate public health policies with individualized cardiometabolic risk management.

Alongside the epidemiological dimension, several contributions to the Research Topic examine the role of metabolic alterations in the pathogenesis of CKD. Liao et al. identified an independent, non-linear association between the triglyceride/HDL cholesterol ratio (TG/HDL-c) and the risk of CKD, confirming the role of lipid metabolism disorders, insulin resistance, and inflammation in renal vulnerability. Huang et al. report that dietary acid load is associated with several indices of insulin resistance in patients with CKD. The link between metabolism and muscle alterations was also investigated by Zhang et al., who observed a positive association between the triglyceride-glucose index (TyG index) and sarcopenia in patients with CKD, confirming that dysfunction of insulin signalling pathways in skeletal muscle interferes with muscle metabolism and protein synthesis processes. The role of the extracellular matrix in sarcopenia associated with type 2 diabetes, as described by Sun et al., highlights how tissue remodelling can contribute to muscle deterioration and insulin resistance. Collectively, these studies support the concept that CKD progression is closely intertwined with systemic metabolic dysfunction, particularly insulin resistance, lipid abnormalities, and alterations in skeletal muscle metabolism. The emerging links between metabolic indices and renal outcomes further emphasize the systemic nature of CKD, which extends beyond the kidney to involve broader cardiometabolic and musculoskeletal pathways. Understanding these interconnected mechanisms may help identify high-risk phenotypes and support earlier preventive interventions.

Other interesting topics emerge from the Research Topic contributions, such as the identification of biomarkers useful for early diagnosis and risk stratification in patients with CKD. The serum uric acid-to-albumin ratio (sUAR), studied by Du et al., appears to be an independent predictor of diabetic nephropathy in patients with type 2 diabetes, as it is an integrated indicator of systemic inflammatory and metabolic status. Sun et al. identified the Ins60/ApoA ratio as a potential early biomarker of diabetic kidney disease in patients with newly diagnosed diabetes, reinforcing the concept that early modulation of metabolic factors may be a key strategy for slowing CKD progression. Furthermore, Fu et al. demonstrated that elevated levels of complement C3 are associated with vascular calcification in patients with non-dialysis CKD, highlighting the importance of inflammation as a modifiable factor in CKD progression and associated cardiovascular risk. The role of inflammation is further supported by the study by Hou et al., which showed that integrating inflammatory indices (neutrophil-to-lymphocyte ratio, NLR; platelet-to-lymphocyte ratio, PLR; lymphocyte-to-monocyte ratio, LMR), metabolic (glucose-to-lymphocyte ratio, GLR) and nutritional (albumin, ALB) indices can identify a high-risk phenotype in haemodialysis patients characterised by sustained hyperglycaemia, chronic inflammation and hypoalbuminaemia. Ma et al. demonstrated that the systemic immuno-inflammatory index (SII) is also associated with increased all-cause and cardiovascular mortality in CKD patients, suggesting that excessive inflammatory activation and a concomitant insufficient immune response may contribute to a worse prognosis. Overall, the biomarkers explored in this Research Topic highlight the growing interest in integrated indicators reflecting the interaction between inflammation, metabolic dysfunction, and immune activation in CKD. Rather than relying on single markers, composite indices derived from routine laboratory parameters may offer practical tools for early risk stratification in clinical settings. Such approaches could facilitate a shift toward more personalized monitoring strategies aimed at identifying patients at risk of accelerated renal and cardiovascular complications.

Other contributions in this Research Topic focus on clinical factors and predictive models associated with kidney disease progression. Fu et al. analysed longitudinal patterns of haemoglobin and haematocrit in patients with stage 3–4 CKD, demonstrating that maintaining relatively stable levels of these parameters over time is associated with a lower risk of disease progression. In a different clinical setting, Ye et al. compared renal function trends between cancer and non-cancer patients, highlighting a higher baseline eGFR in the former, likely due to an inflammatory and hypermetabolic state, and a consequent marked decline in renal function over time. Zhu et al. developed a nomogram for predicting the risk of acute kidney injury (AKI) in patients with ischaemic stroke undergoing endovascular therapy, based on largely modifiable determinants such as hypertension, smoking, proteinuria, blood glucose on admission, serum creatinine, and duration of mechanical ventilation. These findings underscore the importance of longitudinal clinical data and predictive modelling in improving risk assessment for renal outcomes across different clinical contexts. Integrating dynamic clinical variables with traditional risk factors may enhance the ability to detect early trajectories of renal decline and guide preventive interventions. In this perspective, predictive models represent an important step toward more proactive and individualized management of patients at risk of kidney disease progression.

The Research Topic also includes contributions on the pathophysiological mechanisms and potential therapeutic strategies for diabetic nephropathy and other renal diseases. SGLT2 inhibition may attenuate diabetic tubulopathy by suppressing SGK1-mediated pyroptosis, as established by Shi et al.. Similarly, Du et al. conducted a systematic review and meta-analysis on the efficacy of stem cell therapy in patients with diabetic kidney disease, with potential effects on the modulation of inflammation, apoptosis, and renal fibrotic processes. However, as highlighted in the commentary by Xu et al., clinical translation of these strategies remains complex due to the limited methodological quality of available studies and the heterogeneity of experimental protocols. Finally, Li et al. reported a case of coexistence of anti-glomerular basement membrane disease and membranous nephropathy, highlighting the diagnostic complexity of concomitant glomerular diseases. Together, these contributions highlight the evolving therapeutic landscape of kidney disease, ranging from mechanistic insights into metabolic pathways to emerging regenerative approaches. While experimental findings offer promising targets for intervention, translating these strategies into clinical practice will require rigorous validation and standardized study designs. Future research should aim to bridge the gap between experimental discoveries and clinical application in order to develop more effective therapies for slowing CKD progression.

This Research Topic offers a comprehensive overview of the factors contributing to the progression of CKD, including epidemiological, metabolic, clinical, and biological determinants. Integrating evidence from epidemiological studies, emerging biomarkers, predictive models, and new therapeutic strategies will enhance our understanding of the mechanisms underlying renal disease progression and support the development of more effective approaches for the prevention and management of CKD.

We express our gratitude to all the authors who contributed to this Research Topic, as well as to the reviewers and editorial team for their dedication to maintaining the scientific quality of the published works. We hope this Research Topic will stimulate further interdisciplinary collaborations and encourage new research aimed at improving the prevention, diagnosis, and treatment of chronic kidney disease (**Figure 1**).

**Figure 1:**
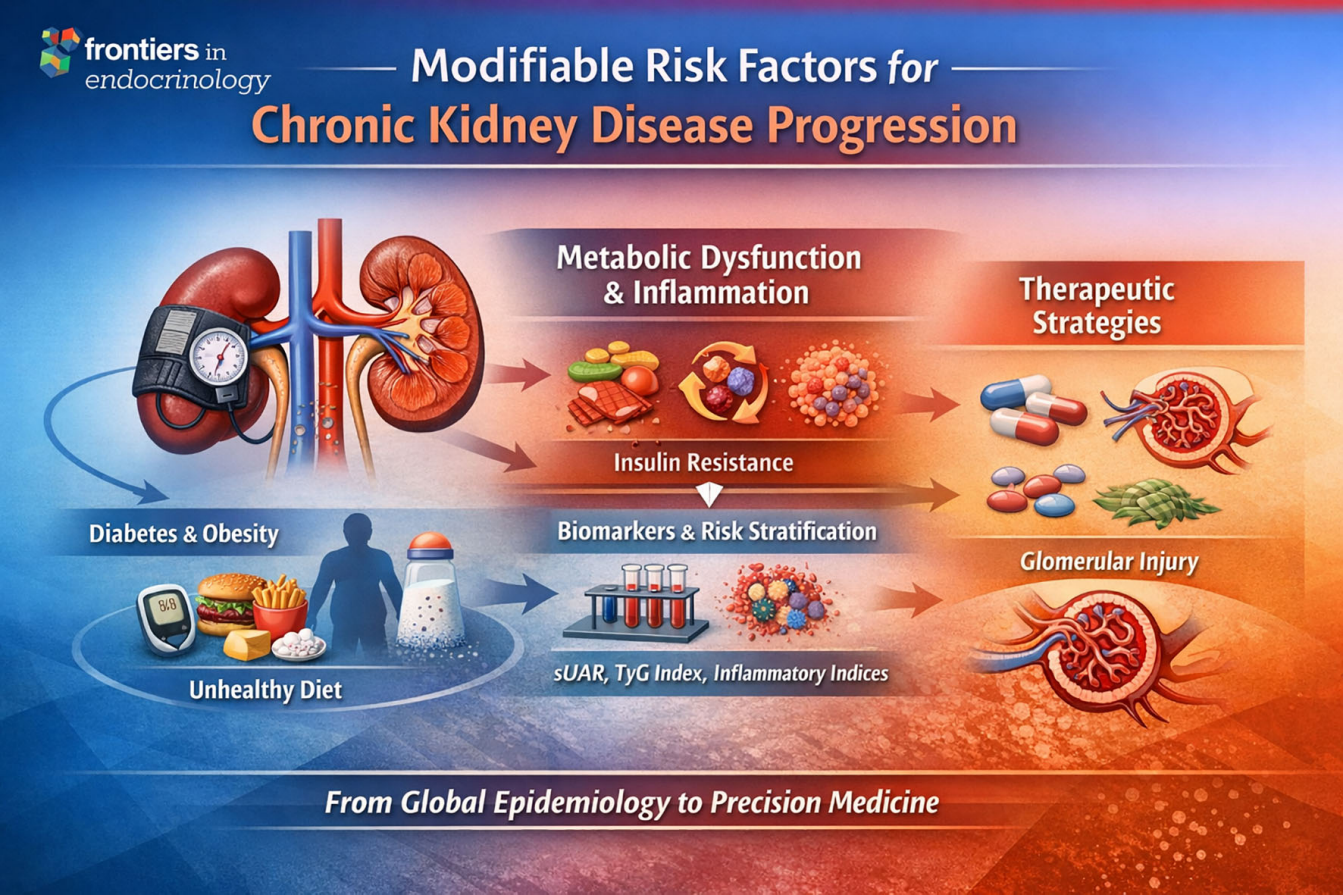
Conceptual framework of modifiable determinants involved in chronic kidney disease (CKD) progression. This schematic overview summarizes the main themes emerging from the Research Topic, illustrating how modifiable cardiometabolic risk factors—including hypertension, diabetes, obesity, and unhealthy dietary patterns—contribute to CKD development and progression. These factors promote metabolic dysfunction, insulin resistance, and chronic inflammation, which in turn drive renal structural damage and functional decline. The figure also highlights the growing role of integrated biomarkers and predictive indices in improving risk stratification, as well as emerging therapeutic strategies aimed at targeting metabolic and inflammatory pathways. Together, these elements illustrate a translational continuum linking global epidemiological determinants with precision medicine approaches for the prevention and management of CKD.

## References

[1] Wu S Si T Shi Z Wang L Song Y Qin L . Trends in the disease burden of chronic kidney disease and anemia attributable to chronic kidney disease in China, 1990-2021. Eur J Med Res. (2026). doi: 10.1186/s40001-026-04033-4, PMID: 41703650

